# The first appearance of the zoonotic parasite *Cryptosporidium mortiferum* in human and tree and ground squirrels in Central Europe

**DOI:** 10.1080/22221751.2025.2456148

**Published:** 2025-01-17

**Authors:** Martin Kváč, Alena Konvalinová, Nikola Holubová, Zuzana Hůzová, Marta Kicia, John McEvoy, Kristina Beranová, Bohumil Sak

**Affiliations:** a Institute of Parasitology, Biology Centre of the Czech Academy of Sciences, České Budějovice, Czech Republic; b Faculty of Agriculture and Technology, University of South Bohemia, České Budějovice, Czech Republic; c Czech Union of Nature Conservationists Animal Rescue Center, Vlašim, Czech Republic; d Institute of Public Health based in Ústí nad Labem, National Reference Laboratory for the Diagnosis of Parasitic Intestinal Diseases, Ústí nad Labem, Czech Republic; e Department of Biology and Medical Parasitology, Wroclaw Medical University, Wroclaw, Poland; f Department of Microbiological Sciences, North Dakota State University, Fargo, ND, USA

**Keywords:** *Cryptosporidium mortiferum*, human, squirrels, infection

## Abstract

*Cryptosporidium mortiferum*, a parasite transmitted by squirrels, is beginning to spread in Europe. *C. mortiferum* was detected in a symptomatic human. A 44-year-old man from the Czech Republic suffered from gastroenteritis characterized by abdominal pain, nausea, and loose stools. Molecular analyses confirmed the XIVaA20G2T1 subtype in the patient's stool. At the same time, the same subtype of *C. mortiferum* was detected in three red squirrels and two ground squirrels in the area where the patient lived. The intensity of the infection was significantly higher in the red squirrels that died, while the ground squirrels showed no symptoms. The results of the study indicate that red squirrels and ground squirrels are the reservoirs for the infection.

*Cryptosporidium* is a globally distributed protist parasite that infects wild, farm, and domestic vertebrates as well as humans. Most infections lead to gastrointestinal disease characterized by diarrhea. While *C. parvum* and *C. hominis* are still the predominant pathogens in humans, there has been an increase in the number of rodent-borne *Cryptosporidium* infections, including those caused by *C. mortiferum* [1], which commonly parasitizes tree squirrels, chipmunks, and deer mice in North America [2]. First human infection has been reported in 1999 in the USA (AF133842). At the beginning of the twenty-first century, cases of *C. mortiferum* infection in humans were only reported in the USA, but in the last 5 years 70 human cases have been reported in Sweden, Denmark, Finland and France [3–6].

In Europe, *C. mortiferum* was first documented in Eurasian red squirrels in Italy in 2008, and its presence was linked with the introduction of the eastern gray squirrel to Italy in the mid-twentieth century [7, 8]. However, apart from natural infections of gray squirrels in Italy and some natural infections of red squirrels in Scandinavia, there are no other data on the spread of this parasite in Europe by wild animals. Furthermore, it is not entirely clear how *C. mortiferum* entered Scandinavia, as eastern gray squirrels are not documented as a natural source of *C. mortiferum* in this part of Europe [5, 6]. This report is another part of the story about the spread of *C. mortiferum* in Europe. Our surveillance results have shown the occurrence of *C. mortiferum* in human, red squirrel and European ground squirrels in the Czech Republic.

The Institute of Public Health in Ústí nad Labem, Czech Republic, regularly collects human stool samples that tested positive for *Cryptosporidium* by microscopy or qPCR from healthcare facilities and sent them to the BC CAS Institute of Parasitology for molecular genotyping. Concurrently, fecal samples from ground and tree squirrels from rescue centres are regularly examined for oocysts and *Cryptosporidium-*specific DNA. These separate projects were linked by the detection of *C. mortiferum* in human and squirrels.

All samples were examined for the presence of *Cryptosporidium* oocysts by Ziehl-Neelsen staining and light microscopy at a magnification of 1,000× [9], by PCR/sequencing and qPCR. Total gDNA from 200 mg of sample and positive control in each series was extracted [10]. *Cryptosporidium* specific nested PCR was used to amplify the small-subunit (SSU) rRNA and *Cryptosporidium* 60-kDa glycoprotein (*gp60*), actin, *Cryptosporidium* oocyst wall protein (*COWP*), 70-kDa heat shock protein (*HSP70*), thrombospondin-related adhesive protein of *Cryptosporidium*-1 (*TRAP-C1*) genes [2, 11, 12]. DNA of *C. serpentis* and *C. mortiferum* XIVaA18G2T2 were used as positive controls; ultrapure water was used as a negative control. PCR products were evaluated by gel electrophoresis, purified by GenElute™ Gel Extraction Kit (Sigma, USA), and sequenced in both directions (SeqMe, Czech Republic). Nucleotide sequences were edited manually using the programme ChromasPro 2.1.4 (Technelysium, South Brisbane, Australia) and Neighbor-joining analyses were computed in MEGA X software (http://mafft.cbrc.jp/alignment/software/). Infection intensity was determined as SSU gene copy number (GCN) using the *Cryptosporidium* qPCR kit YSL-qP-EC-Crypto-100 (YouSeq Ltd, UK). Histological sections of the gastrointestinal tract of red squirrels infected with *Cryptosporidium* were stained with periodic acid-Schiff. The study, conducted with anonymized samples, did not require patient consent. Fecal samples from squirrels were collected during routine trapping and health screening at rescue centres.

In April 2023, a 42-year-old man living in an urban area in Central Bohemia (Figure 1) suffered from gastroenteritis characterized by abdominal pain, nausea, and loose stools. His stool sample tested negative for gastrointestinal bacteria (*Salmonella*, *Shigella*, *Campylobacter*, *Yersinia*, and *Clostridium*) and parasitic protists (*Encephalitozoon, Enterocytozoon, Giardia, Blastocystis* and *Cyclospora*), but positive for *Cryptosporidium* sp. by modified Ziehl – Neelsen staining and qPCR with infection intensity of 588,292 GCN. Molecular/sequencing analysis revealed the presence of *C. mortiferum* of all tested genes, subtype XIVaA20G2T1 (Figure 1). The patient, who had not travelled abroad recently (30 days) and had no direct contact with wild animals but frequented forest paths, was treated symptomatically and recovered after six days.

**Figure 1.**
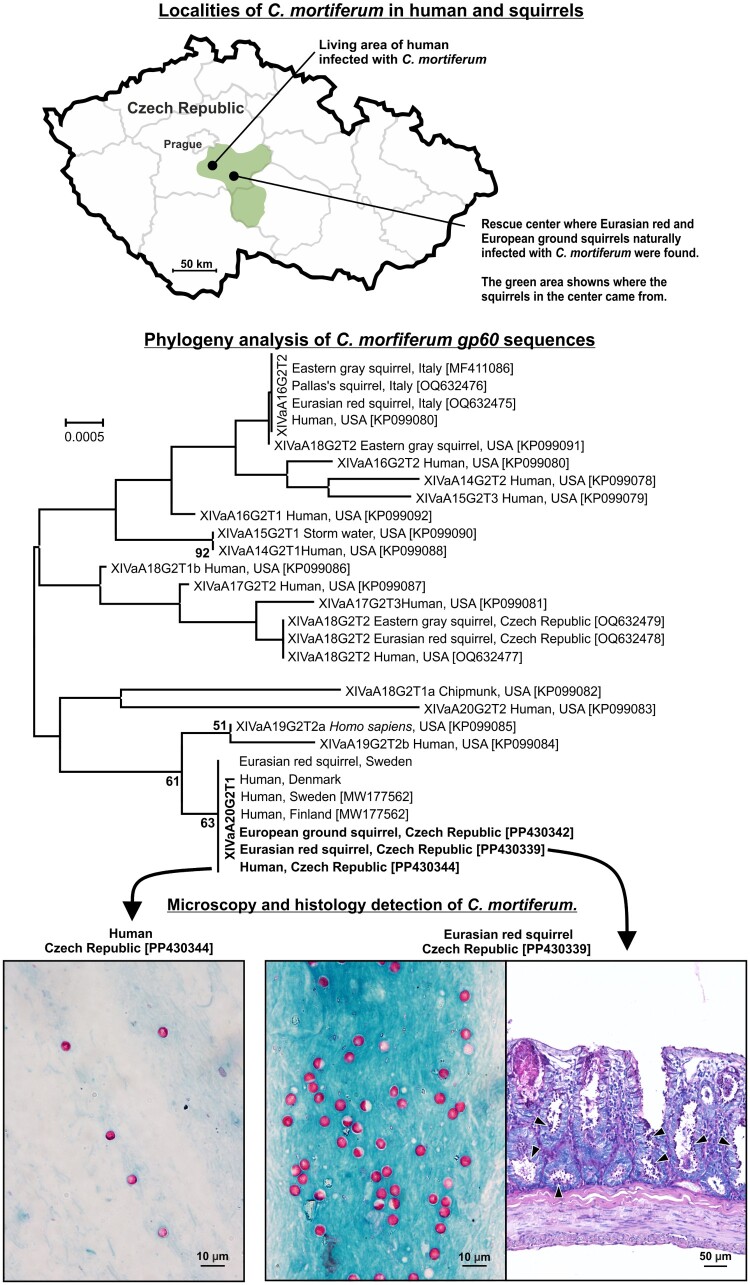
Localization of *Cryptosporidium mortiferum* in human, Eurasian red and European ground squirrels. Phylogenetic analysis of *C. mortiferum gp60* DNA sequences from a 42-year-old immunocompetent male, red and ground squirrels (highlighted). Presence of oocysts stained by Ziehl-Neelsen and histological sections of the caecum from a naturally infected red squirrel that died in a rescue centre. Developmental stages indicated by arrowhead.

Of carcasses from 68 red squirrels and 25 adult ground squirrels kept at rescue centres, three subadult red squirrels and two adult ground squirrels from Czech rescue centres tested positive for *C. mortiferum*-specific DNA by PCR and qPCR, with infection intensities between 772,316 and 1,025,348 GCN in red squirrels and 23,647–28,000 in ground squirrels. Feces from red squirrels were also microscopically positive (Figure 1). Sequence analyses on screened genes showed the subtype of *C. mortiferum* in all squirrels matched the human case. All three red squirrels positive for *C. mortiferum* died of diarrhea and apathy within 10 days of admission to the rescue centre, showing symptoms similar to those in experimentally infected red squirrels [13]. Conversely, ground squirrels showed no signs of cryptosporidiosis, consistent with mild or no symptoms in experimentally infected laboratory rodents and gray squirrels [12, 14].

Seventeen subtypes of *C. mortiferum* have been identified, with most specific to North America. Infections in Central and Northern Europe are caused by subtypes XIVaA20G2T1 and XIVaA19G2T1, not found elsewhere [13]. The results of this and other studies indicate an increasing number of infections with *C. mortiferum* in humans and animals in Europe [3, 14]. The increasing number of infections in Europe indicates direct and indirect transmission from animals to humans [3, 5, 6, 14]. Gray squirrels have not been found in Central and Northern Europe, suggesting the spread of *C. mortiferum* in red squirrels might pose an infection risk to humans [5, 13, 14]. The detection of asymptomatic infections in ground squirrels suggests the parasite could be present in other wild rodent hosts, as seen in North America, and can serve as a source of infection [4].

The identification of the *C. mortiferum* in a human and local wildlife highlights the potential for indirect transmission from wild animal reservoirs to humans. Based on our findings we conclude that *C. mortiferum* emergend in Central Europe and tree nad ground squirrels contribute to its spread.
